# Building and executing a research agenda toward conducting implementation science in medical education

**DOI:** 10.3402/meo.v21.32405

**Published:** 2016-08-25

**Authors:** Patricia A. Carney, Gerald E. Crites, Karen H. Miller, Michelle Haight, Dimitrios Stefanidis, Eileen Cichoskikelly, David W. Price, Modupeola O. Akinola, Victoria C. Scott, Summers Kalishman

**Affiliations:** 1 Department of Family Medicine, Oregon Health & Science University School of Medicine, Portland, OR, USA; 2 Department of Public Health & Preventive Medicine, Oregon Health & Science University School of Medicine, Portland, OR, USA; 3 Department of Medicine, Augusta University/University of Georgia Medical Partnership, Athens, GA, USA; 4 School of Medicine, University of Louisville, Louisville, KY, USA; 5 Department of Pediatrics, Oregon Health & Science University School of Medicine, Portland, OR, USA; 6 Department of Surgery, Carolinas Healthcare System, Charlotte, NC, USA; 7 Department of Family Medicine, College of Medicine, University of Vermont, Burlington, VT, USA; 8 American Board of Medical Specialties Research and Education Foundation, Chicago, IL, USA; 9 Department of Family Medicine, University of Colorado School of Medicine, Denver, CO, USA; 10 Department of Pediatrics, Wake Forest School of Medicine, Winston-Salem, NC, USA; 11 Department of Psychology, University of North Carolina, Charlotte, NC, USA; 12 Department of Family and Community Medicine, University of New Mexico, Albuquerque, NM, USA

**Keywords:** best educational practices, curriculum development, team-based care, educational research, undergraduate/graduate medical education

## Abstract

**Background:**

Implementation science (IS) is the study of methods that successfully integrate best evidence into practice. Although typically applied in healthcare settings to improve patient care and subsequent outcomes, IS also has immediate and practical applications to medical education toward improving physician training and educational outcomes. The objective of this article is to illustrate how to build a research agenda that focuses on applying IS principles in medical education.

**Approach:**

We examined the literature to construct a rationale for using IS to improve medical education. We then used a generalizable scenario to step through a process for applying IS to improve team-based care.

**Perspectives:**

IS provides a valuable approach to medical educators and researchers for making improvements in medical education and overcoming institution-based challenges. It encourages medical educators to systematically build upon the research outcomes of others to guide decision-making while evaluating the successes of best practices in individual environments and generate additional research questions and findings.

**Conclusions:**

IS can act as both a driver and a model for educational research to ensure that best educational practices are easier and faster to implement widely.

It takes an estimated 17 years to get healthcare practices proven to be effective into day-to-day clinical practice (1), which delays offering patients the best possible care. Implementation science (IS) was developed to optimize quality of care by narrowing the gap between research and practice (2). It involves the scientific study of methods to promote the uptake of research results and evidence-based practices to improve the quality and effectiveness of health services (3). Specifically, IS seeks to clarify what interventions work where, when, how, and for whom to implement innovations, programs, and processes effectively (3). Recent healthcare reform legislation has heightened awareness of the promise IS has toward reforming our educational systems and improving healthcare outcomes (4). By incorporating IS into the repertoire of learning resources, medical educators are better able to design programs and systems that enhance educational outcomes of our learners.

Similar to healthcare delivery systems, educational institutions that train health professionals are highly complex (5). These two types of organizations are typically co-mingled, with many healthcare organizations serving as clinical training centers for medical students, residents, and other allied health professionals. Lessons learned from the application of IS in the clinical environment can inform the application of IS in other health professions education settings. Price et al. (6) recently published an article on the implications that IS has for medical education, including that it can help schools achieve changes in learner performance and competence as well as patient outcomes. These authors also suggest that IS should be incorporated into curricula across disciplines and the health educational continuum to further facilitate achievement of implementing best educational practices. For example, O'Flaherty and Phillips (7) tested their own use of the flipped classroom strategy (independent preparation followed by in-class complex group learning tasks (8)). They did this by conducting a literature review to identify best practices, and then developed research questions related to their own institution and used these to conduct an assessment of strategies, evaluations, and outcomes.

These broad views are important and invaluable, but what is missing from the literature on IS in medical education is how best to generate an IS research agenda and identify processes for overcoming typical challenges. Using practical examples on how IS can be applied in diverse educational settings will assist educational leaders, educators, and educational researchers foster IS in their learning settings.

In this article, we use a scenario-based educational example to describe how to apply a structured IS approach to overcome implementation challenges in medical education, and we identify additional educational research questions that IS approaches can help address. The scenario is applicable to undergraduate medical education, graduate medical education, and interprofessional medical education, which we purposefully selected, given the importance of team-based care in the health professions. Finally, we suggest next steps to advance the IS research agenda across the medical educational continuum. While our intention is to target educators and educational researchers in this work, it will become evident that collaboration among researchers, educators, and educational leaders as well as many other stakeholders is necessary for IS to be successful and beneficial for all.

## Research questions that IS can help address

Best Evidence Medical Education (BEME) is an international group committed to the development of evidence-informed education in medicine and other health professions (9). Table 1 provides examples of questions we identified based on effective educational practices identified by BEME. It is a useful guide for research questions that, if addressed, could produce effective educational outcomes. Many important questions exist about how to more effectively incorporate best educational practices into learning settings. We organized a set of such examples into three thematic areas: 1) instructional design and teaching process; 2) facilitators and barriers to implementation of best or proven practices; and 3) costs, timing, and policy-related issues associated with successful adoption for medical schools and programs across the educational continuum.

**Table 1:** Research questions that implementation science can help address

Implementation science thematic area	Example IS research questions based on existing best evidence from BEME
Instructional design and teaching processes	What facilitators can be employed to rapidly transform instructional design to include techniques that optimize working memory use or cognitive load of medical student learners? Which strategies are most effective for implementing interprofessional education across several institutions (Schools of Medicine, Nursing and Allied Health)? How can effective high fidelity medical simulators be used across health professions schools? How can more effective approaches to giving and getting feedback be incorporated into clinical precepting?
Identifying what facilitates or hinders implementation of best practices	What barriers and facilitators exist in incorporating best educational practices in undergraduate, residency, and continuing medical education activities? What factors hinder the development of effective self-monitoring and reflective practice? What approaches are best to undertake or avoid in developing e-learning portfolios? What factors affect successful adoption of electronic health records in physician training?
Costs, timing, and policy-related issues that are associated with successful adoption for medical schools and programs across the educational continuum	What is the cost of adopting interprofessional training? Has the implementation of the ACGME competencies in residency education led to improved resident education and patient outcomes? What are the organizational/institutional cost of IS in medical education? How can multi-institutional studies on the implementation of best educational practices be promoted and funded? How can organizational strategies to adopt IS be improved? What are the costs, policies, regulation, and adaptive institutional responses that enable best IS adoption and implementation?

**Fig. 1:**
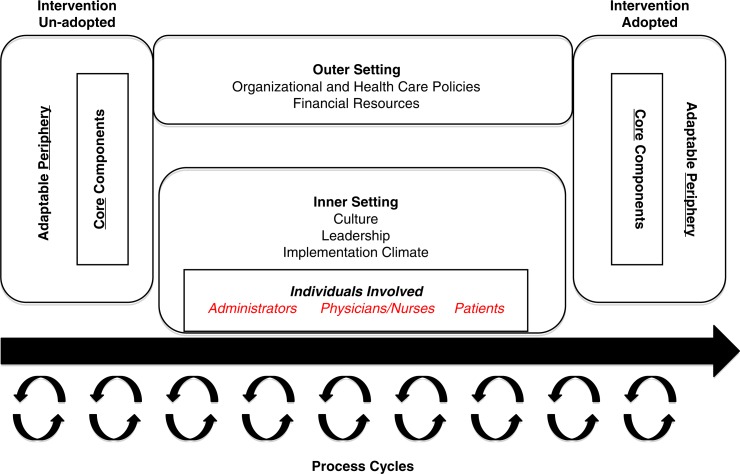
Consolidated framework for implementation research (CFIR).

**Fig. 2:**
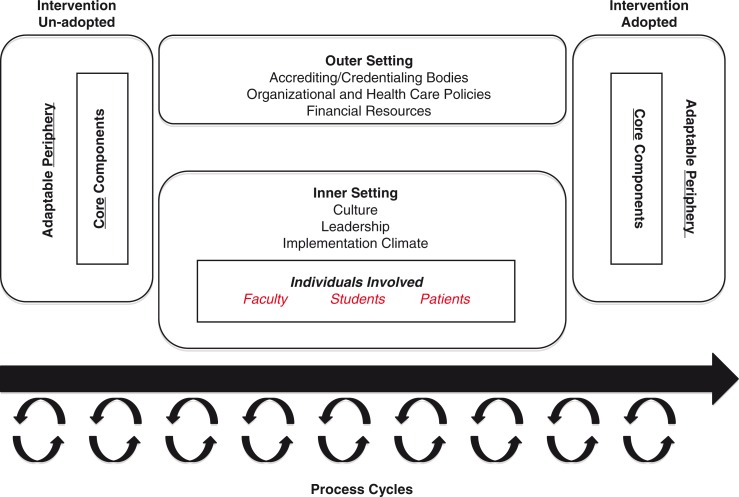
Adapted conceptual framework for implementation research in medical education.

As with other research endeavors, IS for medical education research should be guided by an appropriate framework. While many theories, models, and frameworks exist for IS (10), the specific ones applied to IS research provide the context in which to examine why an implementation succeeds or fails (10). Because the focus of this article is on studying IS in medical education toward understanding and/or explaining influences on implementation outcomes, we have chosen Damschroder et al.'s consolidated framework for implementation research (CFIR). The CFIR (Fig. 1) (11) has become a popular framework for guiding the study of successful implementation within healthcare systems, as it assists in determining how components might be modified to help an innovation move from adoption failure (left side of model) to success (right side of model) (11). In the three-part example below, we apply the concepts of CFIR (Figs. 1 and 2) to different categories of research questions in medical education (Table 1) that IS can help to address.

### Example scenario

Over utilization of the emergency department (ED) is a long-standing problem for university hospitals, and implementation of the Patient Protection and Affordable Care Act (PPACA) has increased the use of the ED by underserved populations (12). A current challenge in university hospitals as well as community-based settings is how to educate a diverse population on how and when to use community-based clinics versus when to use the ED.

#### Thematic area 1: instructional design and teaching processes

Example 1

*Education strategy.* A first-line strategy would be to train medical, nursing, and allied health students, residents, and nurse practitioner trainees simultaneously with faculty and other health professionals on how to better educate patients about community care compared to ED care and how making the best choice at the right time will benefit both patients and their families.

*Research question.* Which strategies are most effective for implementing interprofessional education across several disciplines (e.g., medicine, nursing, and allied health) within our institution? CFIR asks us to define the entire population of individuals involved. It further emphasizes the need for understanding the culture.

- To approach this question, use current literature and structured discussions with faculty and learner stakeholders to identify proven practices to teach across multiple professions at the same time.
  For example, in 2007, BEME published a systematic review on best Search Results interprofessional education (IPE) practices (13), which revealed that faculty development has an important influence on the effectiveness of IPE for learners who have unique values. Customization and authenticity are important mechanisms for positive outcomes of IPE, which has been shown to enhance the development of practice and improvement of services (13). These principles should be implemented in this IPE training program, and IS evaluation strategies should be included to understand how this program was both successfully implemented as well as to identify the barriers that had to be overcome, so the study of further implementation practices can be improved. In the ED scenario, the BEME recommendations would need to be adapted to understand the different learning discipline requirements, focusing on each person's role for educating patients and managing care transitions.

It is interesting to note that overuse of the ED was also selected by Lynch et al. (14) as the topic for a 2015 interdisciplinary workshop using IS to address a real-world problem.

Example 2

*Research question.* How can providing feedback to all care team learners be effectively incorporated into clinical work?

- Because the CFIR model is cyclical, it requires mechanisms in place to assess and evaluate the intervention to continually improve processes. In this case, feedback is a primary mechanism for assessment. As the patient education skills in this scenario are taught and applied, using feedback from peers, attendings, other professional staff, and patients will help with revisions and improvements to the training process, the individual skills of faculty and learners, and will theoretically improve patients’ appropriate use of the ED.

For example, Clynes and Raftery (15) found inconsistencies in the provision of feedback to students that were related to inadequate supervisor training, an unfavorable learning environment, and insufficient time spent with students.

This study indicates that preceptors need effective training, including an appreciation of the steps of the feedback process, an understanding of the student response to feedback toward developing effective communication skills (15). Given that benefits of feedback include increased confidence, motivation, and self-esteem on the part of the learner, as well as improved clinical practice, implementing effective programs and studying them to ensure they are fully in place is vitally important in many educational settings. In our scenario, using the ‘failure’ data from this research report, the educational leaders involved in patient education and transitions in the ED should meet to plan feedback sources and processes, such as identifying which sources of data (e.g., patient surveys, ED readmission, and literacy improvements) are most useful for feedback data. They should also work with learners to make sense of the data and how to effectively interpret the meaning of various feedback processes. These steps will likely identify additional research questions to pose and address.

Tavakol et al. (16) help by differentiating between medical education evaluation and medical education research: ‘Evaluation provides an overview of medical education issues; research is a biopsy of medical education practice’. But both evaluation and research can benefit from a well-founded, structured approach.

#### Thematic area 2: identifying facilitators and barriers for implementation of best educational practices?

Example 1

*Educational strategy.* Create a shared understanding for patients’ frame of reference by determining *why* patients select ED care rather than neighborhood clinic care. As part of the CFIR model that identified patients as a key stakeholder, processes were needed to include their voices. In doing so, an understanding can be gained regarding both the inner and outer settings of the implementation.

*Research question.* What is the public's perception of the role the ED plays in their own healthcare, and what training models can help address misperceptions?

- In 2013, Shaw et al. (17) conducted a study on the decision-making process of patients who use the ED for primary care needs. Their findings are displayed as a decision-making flow chart that could be extremely useful in identifying decision points where instructional interventions could be most effective. In another study by Koziol-McLain et al. (18), patients reported that the stress in their lives had influenced their perceptions that they needed emergency care for non-urgent medical problems. In our scenario, the educational leaders in the ED should ask how the new care processes can be taught to learners and how learners can educate the ED system when flaws in the process are found. In addition, including an assessment of patients’ perspectives and motivations for seeking care and having health professionals educate patients about other non-ED options that are better suited to their healthcare problems would likely reduce overuse of the ED. Studying the effectiveness of implementing these new processes will inform this organization on what works.

Example 2

*Research question.* What factors hinder the development of self-monitoring and reflective practice regarding interprofessional communication?

- Self-monitoring includes all stakeholders; the ED department example includes learners, educators, and patients. However, the development of informed self-monitoring (being aware of one's cognitive and emotional strengths and weaknesses) and reflective practices (ability to reflect on recent learning experiences and during learning experiences) can be challenging (19, 20).

For example, a hallmark paper by Boud underscores the importance of context and locating learning in both educational and professional practice for reflective practices and reflecting to be enhanced (19). Yet in the ED example, the context creates challenges for learners from different disciplines to work collaboratively, given the time barriers in this environment (e.g., shifts, attendance to other learning activities). Thus, a focus on which methods of communication would enhance learners’ cross-discipline discourse would be effective given the time barriers.

#### Thematic area 3: costs, timing, and policy-related issues that are associated with successful adoption for medical schools and programs across the educational continuum

Example 1

*Education strategy.* The CFIR model includes attention to financial resources, which can serve as barriers or facilitators for adopting an intervention. An initial step would be to identify a cost analysis model that meets institutional needs. In this case, it would likely include both real costs, such as training materials, and hidden or incidental costs, such as faculty time and curricular time, which can be less precise.

*Research question.* What is the cost of adopting this interprofessional training program?

- Cost analyses are best conducted when health economists are included to ensure the model chosen matches the activities undertaken and to ensure the outcomes of the enterprise are included in analyses so a cost-to-benefit ratio can be included. Careful planning would need to occur for this type of analysis to produce meaningful results.

A caveat regarding educational research in the health professions, IS, or other types of educational research is that funding sources are limited (21), but the following strategies may help:

- Systematic reviews, literature searches, professional advice, information from professional organizations, and grant searches would provide insights into approaching this research question. Only small-to-moderate funding dollars are available through a variety of sources including medical specialty associations and private foundations. Medical educators must be advocates for support needed to move medical education research from having limited influence on practice to one able to generate sound insights regarding actual practices in education and improved health.

In this case, the cost–benefit study may indicate potential long-term savings, as patients choose the most appropriate healthcare delivery site. Such savings could potentially off-set costs, which could drive institutional support. Any implementation project should keep track of incurred costs and savings that will both prove its effectiveness and support its sustainability. Without strong evidence, it is less likely that effective practices that can reduce costs could be successfully implemented. Many additional questions would likely emerge as part of this work.

Example 3

*Research question.* How can the adoption of IS research in medical education be improved?

- When new programs undergo the truly rigorous research supported by IS, they create a history of success that encourages administrators to support additional IS projects. However, according to Rogers (22), some changes are destined to occur more slowly than others.
- Educators who use the IS approach (early adopters) (22) can mentor those who wish to adopt these refined methods. The feedback from learners’ discourse about the strengths and flaws of the processes may be generalizable to other situations, and the educational leaders could then ask what scholarly works should come from this, and who would benefit within their educational institution and other educational institutions and how these would be shared.

The tiered approach in this example demonstrates application of IS using the CFIR model via cyclical strategies, new questions, processes, and stakeholders entering the project in a structured way. Actions are often determined by outcomes of the previous steps, so educators are not left to ‘reinvent’ processes that have already been shown to be better practices. Rather than the ‘single question’ approach often applied to medical education research, an IS approach steps back to allow a broad view of multiple questions applied sequentially to generate best practices. And, as we evaluate the application of these best practices in our own environment, we can share our outcomes and conceptual frameworks in order to grow this body of literature in a meaningful way.

The CFIR provides advantages that are currently lacking with existing educational models. Progressive educational models must take into account that advanced learning activities (such as those that occur during clinical education) are situated in complex environments, comprising problem-solving with authentic cooperative activities with social learning as central components (23). Although a few progressive education models have recently been proposed for the complexities of online learning communities, none focus on the complexities of clinical environments (23, 24). Existing theories, such as cognitivist and humanist traditions, focus on individual learning and development but are less informative with social learning in complex environments (24). Although situated learning and communities of practice add elements of learning through doing, they are often focused on the goals of small learning teams, and not how larger contexts influence learning within and across teams, disciplines, and organizations (24). The CFIR model extends these concepts by providing a framework for organizing research questions and research targets, while helping to identify, capture, and analyze the many variables that influence learning in complex environments.

## Discussion

There are several practical advantages for medical educators to adopt the IS approach. First, by basing decisions on findings that others have already generated through careful investigation, we are less likely to repeat mistakes and better able to focus resources on reforms and/or processes that have a higher probability of success. Second, when we support our proposals for change (and the accompanying research to evaluate the success of change) with research, we are more likely to gain administrative support. And finally, when we consider medical education as a scientific discipline and the learning settings in which we educate as our ‘labs’, we enrich our discipline using a unified, coherent approach to design, application, and evaluation of our processes, expanding a vitally needed body of research.

Educational institutions must respond to the needs associated with educational effectiveness, just as healthcare organizations and clinicians must respond to the unique needs of patients and populations they serve, such as existing fiscal realities, regulatory bodies, and external competitors or *external pressures* (11). Educational institutions are also influenced by communication and informational systems (*internal mediating factors*), resource limitations, unique cultures of change, and leadership capacity (11). While educators’ choices (*individual influences*) can, individually and collectively, influence innovation adoption, issues such as perceived competence and self-efficacy become relevant for both learners and educators. Finally, features of the innovation itself (*innovation structure*) such as complexity, cost, and processes involved, including planning, execution, and evaluation (*implementation processes*), can affect successful adoption. Bonham and Solomon (25), in examining the relationship of IS to academic medicine, argue that IS is ‘a key component of comparative effectiveness research and essential for evidence-based healthcare reform. This perspective adds an additional application of the IS model’.

BEME has now produced 30 guides summarizing evidence for best educational practices. However, more research is needed both to develop new evidence for best educational strategies and to identify best implementation strategies. For IS to advance in medical education, collaboration among all stakeholders is essential. The target audience for the proposed IS strategies described here is not limited to medical educators. This audience includes implementation scientists, academic and healthcare leaders, patients and communities, and all stakeholders, including payors and purchasers, affected by the implemented changes. In addition, IS can act as a driver and a model for medical education research, which would ensure research findings are easier to implement and more widely disseminated to avoid delays in the adoption of best evidence practices.

We posit that three additional efforts are needed to promote successful IS in medical education. First, we need faculty development in the study and application of IS methods in educational settings, especially for junior faculty who are tasked with learning many new skills, including clinical teaching. Second, funding is needed at the institutional level and beyond to support this work. Current funding to study the implementation of best educational practices is limited, and using IS methods for curriculum development will likely go beyond traditional resource modeling strategies. Finally, processes used for IS need to be guided by appropriate theoretical frameworks to ensure that the social aspects of change are considered and evaluated. Success will depend on multidisciplinary collaborations and knowledge development among physicians, nurses and other health professions’ educators, health services researchers, and educational policy analysts, in many educational institutions across the spectrum of physician education.
